# Exploration of Experiences and Perpetration of Identity-Based Bullying Among Adolescents by Race/Ethnicity and Other Marginalized Identities

**DOI:** 10.1001/jamanetworkopen.2021.16364

**Published:** 2021-07-23

**Authors:** Chardée A. Galán, Lynissa R. Stokes, Nicholas Szoko, Kaleab Z. Abebe, Alison J. Culyba

**Affiliations:** 1 Department of Psychology, University of Pittsburgh, Pittsburgh, Pennsylvania; 2 Department of Pediatrics, University of Pittsburgh School of Medicine, Pittsburgh, Pennsylvania; 3 UPMC Children’s Hospital of Pittsburgh, Pittsburgh, Pennsylvania; 4 Division of General Internal Medicine, Department of Medicine, University of Pittsburgh School of Medicine, Pittsburgh, Pennsylvania; 5 Division of Adolescent and Young Adult Medicine, UPMC Children’s Hospital of Pittsburgh, Pittsburgh, Pennsylvania

## Abstract

**Importance:**

Race-based discrimination represents an ongoing public health crisis in the US, manifested by wide-ranging disparities in youth health, mental health, and violence exposure. However, studies of racial discrimination often neglect experiences of identity-based bullying (IBB) stemming from other marginalized identities, such as gender identity and sexual orientation.

**Objective:**

To examine associations between experiences of IBB based on race/ethnicity/national origin and other social identities and youth health, mental health, and violence outcomes.

**Design, Setting, and Participants:**

This cross-sectional study analyzed responses from an anonymous survey conducted at 13 public high schools in Pittsburgh, Pennsylvania, between October 15, 2018, and October 19, 2018. Participants included in the study were in the 9th through 12th grades. Data were analyzed from October 15, 2020, to February 14, 2021.

**Exposures:**

Experiences of bullying and bullying perpetration based on race/ethnicity/national origin and other social identities (ie, gender identity, sexual orientation, religion, physical or mental disability, immigration status, other unspecified reason).

**Main Outcomes and Measures:**

Youth health (delayed well care; forgone medical care; physical, mental, or emotional limitations), mental health (nonsuicidal self-injury, suicidal ideation), and violence involvement (weapon perpetration or survivorship, fighting, sexual assault, adolescent relationship abuse, experiencing homicide of friend or family member) were assessed using self-reported items modeled on the Centers for Disease Control and Prevention Youth Risk Behavior Survey.

**Results:**

Among 3939 participants, the mean (SD) age was 15.7 (1.3) years; 1380 students (36.3%) identified as Black/African American, 2086 (53.7%) as assigned female at birth, 1021 (32.6%) as belonging to a sexual minority group, and 313 (10.0%) as gender diverse. Among reported social identities, race/ethnicity-based experiences of bullying (375 students [9.5%]) and bullying perpetration (209 students [5.8%]) were the most common. Youth with multiple stigmatized identities experienced even higher rates of experiences of IBB and IBB perpetration. Specifically, the highest rates of IBB were reported by gender diverse Black and Hispanic youth. Experiencing IBB based on multiple stigmatized identities was associated with all outcomes, including delayed well care (aOR, 1.41; 95% CI, 1.20-1.65), forgone medical care (aOR, 1.64; 95% CI, 1.44-1.87), nonsuicidal self-injury (aOR, 2.86; 95% CI, 2.53-3.24), suicidal ideation (aOR, 2.49; 95% CI, 2.20-2.83), and greater violence involvement (experiencing violence: aOR, 2.90; 95% CI, 2.45-3.43; homicide survivorship: aOR, 1.19; 95% CI, 1.06-1.33).

**Conclusions and Relevance:**

These results further encourage the development of youth health, mental health, and violence prevention programs that address experiences of bullying based on multiple marginalized identities.

## Key Points

**Question:**

How are experiences of bullying based on race/ethnicity/national origin and other marginalized identities associated with outcomes for health, mental health, and violence among adolescents?

**Findings:**

In this cross-sectional study of 3939 high school youth, the highest rates of experiencing and perpetrating identity-based bullying were demonstrated by youth with multiple marginalized identities. Experiences with and perpetration of bullying based on race/ethnicity were associated with all health, mental health, and violence outcomes examined; experiencing multiple forms of identity-based bullying was associated with worse adjustment.

**Meaning:**

These results suggest that policies that address experiences of race/ethnicity-based bullying and co-occurring mental and physical health outcomes must also attend to other aspects of youths’ social identity.

## Teaser

This cross-sectional survey study of high school students at 13 public high schools in a large US city examines associations of bullying based on racial/ethnic, gender, and other marginalized identities with outcomes for health, mental health, and violence.

## Introduction

Race-based discrimination is an ongoing public health crisis in the US, manifested by wide-ranging disparities in health care access and health outcomes, exposure to community and interpersonal violence, and inequitable criminal justice interactions. Black adolescents experience an average of 5 instances of racial discrimination per day,^1^ underscoring the extent to which anti-Blackness is woven into societal fabric. While Black youth report the highest rates of racial discrimination in the US,^2^ other racial/ethnic minority groups, including Latinx youth, are also frequent targets of race-based discrimination and bullying.^3^ Such experiences during adolescence have been linked to multiple negative emotional, behavioral, and physical health problems,^4,5,6^ which often persist into adulthood.^7,8^ Targeting and responding to adolescents’ experiences with discrimination has the potential to prevent further widening of inequities into adulthood.^9^

Although many racial-ethnic minority youth demonstrate extraordinary resilience by drawing on cultural and familial strengths, repeated encounters with racial discrimination can lead to significant emotional and psychological injury, also known as racial trauma.^10,11^ Other forms of discrimination and identity-based bullying (IBB), including gender-based and ableist bullying, carry similar negative health consequences.^12,13^ IBB can be a form of interpersonal trauma that threatens an individual’s self-esteem and sense of safety, resulting in variable socioemotional responses, including heightened depressive and other internalizing symptoms or increased violence and aggression.^12^ For some adolescents, these expressions of anger may take the form of bullying or harassing others, a behavior conceptualized as the *bully-victim*.^14^ While bully-victims exhibit higher levels of depression, anxiety, and suicidality,^15,16^ the complex mechanisms through which identity affects bullying behaviors remain poorly understood. In particular, traditional models of IBB may neglect broader, interacting systems of oppression that drive these responses.

While studies of single-identity discrimination, including race-based discrimination, have proliferated, research considering IBB based on multiple stigmatized identities is needed. According to intersectionality theory, dynamic structures of power and inequality (eg, racism, sexism, heterosexism) constrain access to resources and shape our lived experience.^17,18^ Thus, other aspects of youths’ sociocultural contexts, such as gender identity, sexual orientation, and immigration status may interact with their race to inform the frequency and types of racial discrimination they experience. In addition, broader societal structures may drive other forms of intersectional oppression, including exposure to interpersonal and community violence.^18^ Together, these identity-based phenomena may factor into how multiply marginalized youth navigate encounters with health care and mental health systems.^9^ Thus, without considering multiple forms of IBB, our ability to meet the diverse needs of racial/ethnic minority youth is limited.

To address this gap, the current study adopted an intersectional approach by jointly considering experiences of discrimination based on race, sexual orientation, gender identity, religion, immigration status, and physical or mental disability. Using a cross-sectional sample of youth, this investigation sought to clarify how different types of IBB independently and collectively affect health, mental health, and violence-related outcomes. Patterns of IBB were examined to elucidate how youth adjustment may differ across various combinations of experiences (ie, experiences of IBB, IBB perpetration, involvement in both).

## Methods

This study sampled 4207 students in grades 9 through 12 at 13 high schools within Pittsburgh Public Schools (PPS), who completed anonymous school-based surveys of health risk and protective behaviors. An informational letter sent to parents/guardians provided an opportunity to opt out of their child’s participation by signing and returning the form. All students who were present in school on the day of survey administration and whose parents/guardians had not opted out were eligible to participate. Students were also given the opportunity to opt out of participation. Questions were modeled on the US Centers for Disease Control and Prevention Youth Risk Behavior Survey (YRBS)^19^ and were administered as a paper-pencil survey during a class period between October 15 and October 19, 2018. Among 6306 students enrolled across all 13 schools, 4487 surveys were collected (a response rate of 71.2%). A total of 280 surveys (6.2%) were excluded—37 surveys were unreadable and 243 were judged too incomplete for analysis. Because data collection was anonymous, we were unable to ascertain demographics of students not completing the survey. This survey was a partnership among PPS, Allegheny County Health Department, and UPMC Children’s Hospital of Pittsburgh. The PPS School Board approved this assessment, and the University of Pittsburgh’s institutional review board deemed this cross-sectional analysis exempt from review because it used deidentified data. No remuneration was provided. This study followed the Strengthening the Reporting of Observational Studies in Epidemiology (STROBE) reporting guideline.

### Measures

#### Racial/Ethnic Identity

Participants were given the following race options and instructed to mark all that apply: White, Black or African American, Asian, American Indian or Alaska Native, Native Hawaiian or Other Pacific Islander, and other. A separate question queried Hispanic/Latino origins. Based on the race and ethnicity prevalence in the sample, we created a race/ethnicity variable with 4 categories that included Non-Hispanic White, Non-Hispanic Black, Hispanic, and multiracial/other.

#### Gender Identity

Gender was assessed with 2 questions about current gender identity and sex assigned at birth. Youth who endorsed their gender identity as transgender, genderqueer, nonbinary, or another identity, and youth whose gender identity differed from their sex assigned at birth were categorized as gender diverse.^20,21^

#### Sexual Orientation

Participants indicated which of the following best describes them by marking yes or no: heterosexual, mostly heterosexual, gay or lesbian, bisexual, queer, asexual, and not sure. Youth who endorsed any response other than heterosexual were coded as sexual minorities.

#### Discrimination and IBB

An experience of IBB was defined as answering affirmatively to being bullied on school property during the past 12 months. To assess experiences of IBB, youth were then asked whether they had been harassed or bullied on school property during the past 12 months for any of the following reasons (yes/no responses): race/ethnicity/national origin, religion, gender, sexual orientation, physical or mental disability, immigration status, and/or other reason.

To assess IBB perpetration, youth indicated whether they had harassed or bullied others based on each of the aforementioned 7 identity categories. In addition to looking at endorsement rates for each identity, we also summed participants’ responses to obtain a total number of stigmatized identities, with separate scores computed for experiences of IBB and IBB perpetration. Because few participants endorsed either category of IBB based on more than 2 identities (81 [2.1%] and 30 [0.8%], respectively), total scores were rescored so that more than 2 identities were scored as 2 (ie, 0 indicates 0 identities; 1 indicates 1 identity; 2 indicates ≥2 social identities).

#### Health and Health Care Outcomes

Well-child care was assessed by asking, “When was the last time you saw a doctor or nurse for a check-up or physical exam when you were not sick or injured?” Responses were recoded so that 0 equaled 2 years or less since routine well visit and 1 equaled more than 2 years since routine well visit. Forgone health care was assessed with a single yes or no question: “In the past 12 months, have you ever thought you needed to go see a doctor, nurse, or go to the emergency room BUT did NOT go?” Participants also indicated whether they “are limited in any way in any activities because of physical, mental, or emotional problems” in a yes or no question.

#### Mental Health Outcomes

Nonsuicidal self-injury (NSSI) and suicidal ideation in the past 12 months were assessed using validated items from the YRBS. NSSI was defined as answering affirmatively (1 time or more) to the question: “How many times have you ever hurt yourself on purpose without wanting to die, such as cutting, pinching, scratching, or burning yourself?” Suicidal ideation was assessed with 1 binary item: “Did you ever seriously consider attempting suicide?”

#### Violence Outcomes

Five YRBS items assessed violence-related outcomes within the past 12 months, operationalized as any or none: violence perpetration (threatened or injured someone else with a weapon), exposure to violence (someone threatened or injured you with a weapon), physical fighting, sexual assault (forced to do sexual things you did not want to do), and adolescent relationship abuse (someone you were dating or going out with forced you to do sexual things or physically hurt you on purpose).^19^ Lifetime homicide survivorship was defined as answering affirmatively (ie, 1 or greater) to the question, “How many of your friends and/or family members have been murdered?”

### Statistical Analysis

Analyses were performed using Stata version 16.1 (StataCorp) and proceeded in 4 steps. First, descriptive statistics were computed to examine demographics, rates of IBB experiences and perpetration, and youth health, mental health, and violence involvement. Second, unadjusted logistic regression models were run to test associations between experiences of IBB based on race/ethnicity/national origin and IBB perpetration based on the 7 specified social identities. Third, we computed adjusted mixed-effects logistic regression models to estimate youth health, mental health, and violence outcomes as a function of the total number of stigmatized identities for experiences of IBB and IBB perpetration, and accounting for the clustering of data by school. Last, we jointly examined experiences of IBB and/or IBB perpetration based on race/ethnicity/national origin. Linear combinations of model coefficients were used to calculate adjusted odds ratios (aORs) for each of the following compared with uninvolved youth: experiences of IBB only, IBB perpetration only, and involvement in both forms of IBB. Adjusted mixed-effects logistic regression was used to regress health, mental health, and violence outcomes on IBB involvement type. All logistic regression models were adjusted for age and included a random intercept for school; given our study’s focus on examining IBB, we did not control for other demographic factors such as race, gender identity, or sexual orientation. Statistical significance was set to *P* < .05 in 2-tailed tests.

Of the 4207 youth who completed surveys, 2950 youth did not respond to the IBB items. Closer examination of the data revealed that 2448 of these participants responded no when asked if they had been bullied on school property within the past 12 months, suggesting that these participants interpreted the subsequent IBB subtype questions as inapplicable to them. This is further corroborated by the fact that these 2448 youth responded to the other items immediately before and after the IBB questions. Thus, IBB items were coded as no if youth did not respond and had answered no to the preceding question inquiring about being bullied on school property. Prior work using similar items from the YRBS have used this approach to handle missing data on IBB questions.^12^ This yielded a total sample size of 3939 out of 4207 youth (6.3% missing IBB data). There were no significant differences in demographics (age, race/ethnicity, sex assigned at birth, gender identity, and sexual orientation) between individuals who completed vs did not complete the IBB items. Analyses were limited to the 3939 participants with IBB data.

## Results

Across all 3939 students included in this study, mean (SD) participant age was 15.7 (1.3) years; 1380 youth (36.3%) identified as Black/African American, 2086 (53.7%) as assigned female at birth, 1021 (32.6%) as belonging to a sexual minority group, and 313 (10.0%) as gender diverse (Table 1). Overall, 1505 participants (38.2%) reported experiences of IBB, 972 (27.0%) reported IBB perpetration, and 890 (24.7%) reported both experiences of IBB and IBB perpetration. Experiences of IBB due to race/ethnicity/national origin were the most commonly reported type (375 participants [9.5%]); however, many participants experienced IBB based on another unspecified reason (756 [19.2%]). IBB perpetration showed similar patterns, with highest rates of perpetration based on race/ethnicity/national origin (209 [5.8%]) or another unspecified reason (476 [13.2%]). Youth with multiple stigmatized identities experienced even higher rates of both sides of IBB (Table 2). Specifically, the highest rates of experiences of IBB and IBB perpetration were reported by gender diverse Black and Hispanic youth. The eTable in the Supplement outlines the detailed constellation of IBB reported by youth. Across all participants, the most frequently endorsed health, mental health, and violence-related outcomes included forgone medical care (872 [33.5%]), NSSI (975 [26.3%]), and homicide survivorship (1606 [42.1%]) (Table 1).

**Table 1.** Prevalence of Demographic Characteristics, IBB, and Health, Mental Health, and Violence Outcomes

Characteristic	Valid responses, No.	Youth, No. (%)
Age, mean (SD), y	3929	15.7 (1.3)
Race/ethnicity^a^	3805	
Non-Hispanic		
White		1339 (35.2)
Black		1380 (36.3)
Hispanic		358 (9.4)
Multiracial/other		728 (19.1)
Sex assigned at birth	3888	
Male		1802 (46.4)
Female		2086 (53.7)
Gender identity	3124	
Cisgender		2811 (90.0)
Gender diverse^b^		313 (10.0)
Sexual orientation	3130	
Heterosexual		2109 (67.4)
Sexual minority		1021 (32.6)
Experiences of IBB	3939	
No endorsement		2434 (61.8)
1 stigmatized identity		1277 (32.4)
≥2 stigmatized identities		228 (5.8)
IBB perpetration	3603	
No endorsement		2631 (73.0)
1 stigmatized identity		909 (25.2)
≥2 stigmatized identities		63 (1.8)
Health and health care		
>2 y since check-up or routine well visit	2685	449 (16.7)
Forgone medical care	2605	872 (33.5)
Physical, mental, or emotional limitations	2558	633 (24.8)
Mental health		
Nonsuicidal self-injury	3714	975 (26.3)
Suicidal ideation	3645	761 (20.9)
Violence involvement		
Perpetration	3915	270 (6.9)
Exposure to violence	3913	349 (8.9)
Physical fighting	3812	1167 (30.6)
Homicide survivorship^c^	3813	1606 (42.1)
Sexual assault	3763	567 (15.1)
Adolescent relationship abuse	3571	402 (11.3)

Abbreviation: IBB, identity-based bullying.

^a^ Non-Hispanic White and Non-Hispanic Black answered affirmatively to White or Black or African American racial categorization, respectively, and no to all other race/ethnicity items. The Hispanic category includes any participants who identified as Hispanic regardless of the race they endorsed. Multiracial identity includes youth who selected more than 1 race (and did not endorse Hispanic ethnicity), while other includes youth who self-identified as Asian, American Indian or Alaska Native, Native Hawaiian or other Pacific Islander, or other (and did not endorse Hispanic).

^b^ Transgender, genderqueer, nonbinary, or other gender identity and youth whose gender identity differed from their sex assigned at birth.

^c^ Lifetime homicide survivorship was defined as answering affirmatively (ie, 1 or greater) to the question, “How many of your friends and/or family members have been murdered?”

**Table 2.** Prevalence of IBB by Demographics and Intersectional Identities

Characteristic^a^	Youth, No. (%) (n = 3939)^b^	
	Experiences	Perpetration
Race		
Non-Hispanic		
White	441 (32.9)	222 (18.8)
Black	511 (37.0)	396 (30.1)
Hispanic	155 (43.3)	109 (33.1)
Multiracial/other	327 (44.9)	187 (28.9)
Sex assigned at birth		
Male	679 (37.7)	503 (29.7)
Female	785 (37.6)	432 (23.2)
Gender identity		
Cisgender	935 (33.3)	544 (21.3)
Gender diverse^c^	219 (70.0)	165 (60.4)
Sexual orientation		
Heterosexual	673 (31.9)	429 (22.0)
Sexual minority	500 (49.0)	302 (33.8)
**Intersections between race/ethnicity and gender identity**		
Non-Hispanic White		
Cisgender	348 (30.2)	171 (16.7)
Gender diverse^c^	55 (61.1)	28 (38.9)
Non-Hispanic Black		
Cisgender	284 (33.3)	209 (25.8)
Gender diverse^c^	71 (73.2)	57 (63.3)
Hispanic		
Cisgender	80 (33.2)	49 (22.2)
Gender diverse^c^	34 (77.3)	30 (73.2)
Multiracial/other		
Cisgender	202 (40.1)	102 (22.8)
Gender diverse^c^	38 (66.7)	29 (63.0)
**Intersections between race/ethnicity and sexual orientation**		
Non-Hispanic White		
Heterosexual	245 (29.1)	133 (17.6)
Sexual minority	157 (39.9)	68 (20.2)
Non-Hispanic Black		
Heterosexual	208 (31.4)	167 (26.2)
Sexual minority	155 (51.8)	106 (38.6)
Hispanic		
Heterosexual	62 (32.6)	39 (22.2)
Sexual minority	53 (58.2)	41 (50.0)
Multiracial/other		
Heterosexual	136 (37.6)	72 (22.0)
Sexual minority	106 (54.1)	62 (38.0)

Abbreviation: IBB, identity-based bullying.

^a^ Percentages in this table are calculated based on the total number of youth in a row. For example, among non-Hispanic White youth, 441 (32.9%) endorsed experiences of IBB.

^b^ Experiences of IBB were coded as a binary; youth received a score of 1 if they answered affirmatively to experiences of bullying based on race/ethnicity/national origin, religion, gender, sexual orientation, physical or mental disability, immigration status, and/or another unspecified reason. They received a score of 0 if they denied experiences of bullying for all social identities. A similar approach was adopted for IBB perpetration, which was also coded as a binary but relied on responses to questions querying IBB perpetration based on each of the 7 aforementioned reasons.

^c^ Transgender, genderqueer, nonbinary, or other gender identity and youth whose gender identity differed from their sex assigned at birth.

As summarized in Table 3, youth who reported experiences of IBB based on race/ethnicity/national origin were more likely to also report IBB perpetration due to their race/ethnicity/national origin (OR, 8.97; 95% CI, 6.58-12.21), religion (OR, 6.42; 95% CI, 4.40-9.38), gender (OR, 3.04; 95% CI, 1.83-5.06), sexual orientation (OR, 3.73; 95% CI, 2.24-6.19), physical or mental disability status (OR, 2.64; 95% CI, 1.45-4.79), immigration status (OR, 2.25; 95% CI, 0.50-3.23), and other reasons (OR, 2.25; 95% CI, 1.70-2.99).

**Table 3.** Associations Between Experiences of Bullying Based on Race/Ethnicity and IBB Perpetration

IBB perpetration	Experienced bullying based on race, ethnicity, or national origin, No. (%)		Odds ratio (95% CI)
	No	Yes	
**Any IBB perpetration**			
No	2547 (77.4)	84 (27.1)	9.19 (7.05-11.95)
Yes	746 (22.7)	226 (72.9)	
**Based on race, ethnicity, or national origin**			
No	3166 (96.1)	228 (73.6)	8.97 (6.58-12.21)
Yes	127 (3.9)	82 (26.5)	
**Based on religion**			
No	3206 (97.4)	264 (85.2)	6.42 (4.40-9.38)
Yes	87 (2.6)	46 (14.8)	
**Based on gender**			
No	3220 (97.8)	290 (93.6)	3.04 (1.83-5.06)
Yes	73 (2.2)	20 (6.5)	
**Based on sexual orientation**			
No	3230 (98.1)	289 (93.2)	3.73 (2.24-6.19)
Yes	63 (1.9)	21 (6.8)	
**Based on physical or mental disability status**			
No	3235 (98.2)	296 (95.5)	2.64 (1.45-4.79)
Yes	58 (1.8)	14 (4.5)	
**Based on immigration status**			
No	3251 (98.7)	305 (98.4)	1.27 (0.50-3.23)
Yes	42 (1.3)	5 (1.6)	
**Other reason**			
No	2891 (87.8)	236 (76.1)	2.25 (1.70-2.99)
Yes	402 (12.2)	74 (23.9)	

Abbreviation: IBB, identity-based bullying.

Experiencing IBB based on multiple stigmatized identities was associated with all health, mental health, and violence outcomes examined; the same pattern of findings emerged for IBB perpetration (Table 4). For example, experiences of IBB (aOR, 1.64; 95% CI, 1.44-1.87) and IBB perpetration (aOR, 1.55; 95% CI, 1.30-1.86) were both significantly associated with forgone health care.

**Table 4.** Associations of IBB With Youth Health, Mental Health, and Violence

Outcomes	IBB, aOR (95% CI)^a^	
	Experiences^b^	Perpetration^c^
Health and health care		
>2 y since check-up or routine well visit	1.41 (1.20-1.65)	1.77 (1.44-2.18)
Forgone medical care	1.64 (1.44-1.87)	1.55 (1.30-1.86)
Physical, mental, or emotional limitations	2.03 (1.77-2.34)	1.54 (1.26-1.88)
Mental health		
Nonsuicidal self-injury	2.86 (2.53-3.24)	2.55 (2.18-2.97)
Suicidal ideation	2.49 (2.20-2.83)	2.12 (1.80-2.50)
Violence involvement		
Perpetration	2.37 (1.95-2.85)	4.99 (3.98-6.27)
Exposure to violence	2.90 (2.45-3.43)	4.39 (3.56-5.40)
Physical fighting	1.73 (1.54-1.94)	2.19 (1.89-2.54)
Homicide survivorship^d^	1.19 (1.06-1.33)	1.44 (1.25-1.66)
Sexual assault	2.37 (2.06-2.72)	2.58 (2.17-3.06)
Adolescent relationship abuse	2.61 (2.23-3.06)	3.27 (2.69-3.98)

Abbreviations: aOR, adjusted odds ratio; IBB, identity-based bullying.

^a^ Odds ratio from mixed-effects logistic regression adjusted for youth age and accounting for school-level clustering.

^b^ Experiences of IBB reflect the number of social identities youth targeted in bullying (0 indicates no endorsement of IBB; 1 indicates endorsed IBB based on 1 social identity; 2 indicates endorsed IBB based on ≥2 social identities).

^c^ Similar to experiences of IBB, IBB perpetration is scored on a 0 to 2 scale and reflects the number of social identities participants have targeted others based on.

^d^ Lifetime homicide survivorship was defined as answering affirmatively (ie, 1 or greater) to the question, “How many of your friends and/or family members have been murdered?”

Table 5 reports adjusted associations between the race/ethnicity/national origin IBB involvement types (ie, experiences of IBB only, IBB perpetration only, involvement in both) and health, mental health, and violence outcomes, with no involvement treated as the reference. Compared with no involvement, experiences of IBB tied to race/ethnicity/national origin was significantly associated with going more than 2 years since a routine well visit (aOR, 1.57; 95% CI, 1.05-2.34), forgone medical care (aOR, 1.70; 95% CI, 1.18-2.38), NSSI (aOR, 2.64; 95% CI, 1.98-3.52), suicidal ideation (aOR, 1.65; 95% CI, 1.19-2.29), and all violence-related outcomes (eg, physical fighting: aOR, 1.80; 95% CI, 1.34-2.42; sexual assault: aOR, 1.81; 95% CI, 1.28-2.56) with the exception of homicide survivorship. Compared with no involvement, race/ethnicity/national origin IBB perpetration demonstrated very similar direct associations with all health, mental health, and violence outcomes. When comparing joint involvement in both experiences of IBB and IBB perpetration with no involvement, there were fewer significant differences that emerged, with the exception of greater suicidal ideation and exposure to violence (Table 5).

**Table 5.** Associations Between IBB Groupings and Health, Mental Health, and Violence

Outcomes	IBB group, aOR (95% CI)^a^			
	No involvement	Perpetration only	Experienced IBB only	Involved in both
Health and health care				
Greater than 2 y since check-up or routine well visit	1 [Reference]	2.41 (1.44-4.02)	1.57 (1.05-2.34)	0.97 (0.44-2.13)
Forgone medical care	1 [Reference]	1.99 (1.20-3.32)	1.70 (1.18-2.38)	1.18 (0.63-2.24)
Physical, mental, or emotional limitations	1 [Reference]	0.91 (0.49-1.70)	0.91 (0.60-1.41)	1.83 (0.95-3.53)
Mental health				
Nonsuicidal self-injury	1 [Reference]	2.76 (1.91-3.99)	2.64 (1.98-3.52)	0.96 (0.56-1.65)
Suicidal ideation	1 [Reference]	2.23 (1.49-3.33)	1.65 (1.19-2.29)	3.53 (2.21-5.64)
Violence involvement				
Perpetration	1 [Reference]	5.37 (3.46-8.32)	2.04 (1.31-3.18)	1.33 (0.59-2.98)
Exposure to violence	1 [Reference]	5.38 (3.57-8.11)	2.53 (1.72-3.72)	2.54 (1.39-4.64)
Physical fighting	1 [Reference]	2.40 (1.62-3.55)	1.80 (1.34-2.42)	1.18 (0.72-1.93)
Homicide survivorship	1 [Reference]	1.97 (1.35-2.88)	1.11 (0.84-1.49)	1.04 (0.65-1.67)
Sexual assault	1 [Reference]	3.84 (2.59-5.71)	1.81 (1.28-2.56)	1.41 (0.77-2.58)
Adolescent relationship abuse	1 [Reference]	3.91 (2.48-6.17)	2.11 (1.44-3.10)	1.74 (0.87-3.49)

Abbreviations: aOR, adjusted odds ratio; IBB, identity-based bullying.

^a^ Odds ratio from mixed-effects logistic regression model adjusted for youth age and accounting for school-level clustering. Data in each row come from a single model, with each column representing the aOR for experiences of IBB with or without perpetration involvement compared with the reference group.

## Discussion

Among a school-based sample of youth in urban neighborhoods, the present study examined experiences of IBB and IBB perpetration based on race/ethnicity/national origin and other stigmatized identities. Examining types of IBB provided key insights into the complex interplay between intersecting identities and highlighted the pervasive exposure to race/ethnicity/national origin IBB among youth, particularly among Black and Hispanic youth who identify as gender diverse.^22^ Of note, youth who experienced race-based bullying were also more likely to perpetrate bullying based on multiple identities, highlighting how perceived stigma, minority stress, and systemic disempowerment factors into interactions with peers.^23,24,25^ The present analysis complements recent intersectional approaches using national YRBS data to examine the prevalence of experiences of bullying among youth who identify as belonging to both racial/ethnic and sexual minority groups.^26,27,28^ We extend these findings by examining the types of both experiences of IBB and IBB perpetration (eg, racial/ethnic/national origin, sexual orientation, gender identity) experienced by youth, and find higher endorsed prevalence across multiple types of IBB in using this approach.

Experiences of IBB and IBB perpetration were both associated with all health, mental health, and violence outcomes examined; experiencing multiple forms of IBB was associated with worse adjustment. Notably, compared with uninvolved youth, youth experiencing IBB due to race/ethnicity/national origin were at increased risk for NSSI, suicidal ideation, exposure to violence, sexual assault, and adolescent relationship abuse. These same youth were also more likely to forego medical care (ie, not go to the emergency department when needed) and go more than 2 years since their last routine well visit. Similar results emerged for youth in the race/ethnicity/national origin IBB perpetration only group.

Follow-up studies are needed to elucidate specific barriers to health care utilization among youth who have experienced race/ethnicity/national origin IBB. However, it is reasonable to expect that an important barrier includes the historical and continued mistreatment of Black (eg, Tuskegee syphilis study) and Hispanic communities, which have led to warranted mistrust of health care systems.^29,30^ Although associations between IBB and service utilization may be driven by additional factors, such as family income, parents’ experiences of discrimination, or insurance status, it is critical to recognize how histories of oppression in the medical system and adolescents’ experiences of discrimination may contribute to these disparities. Our findings align with broadly recognized gaps in the receipt of health services for marginalized youth and serve as a call to action to health care professionals to address experiences of race/ethnicity-based bullying and co-occurring health effects through creating inclusive clinical spaces, talking about instances of racist behavior with patients and families, and dismantling structural drivers of persistent inequities.^9,31,32,33,34,35,36^

Data for the present study were collected in 2018, a time in which we saw increases in racist political rhetoric, racial hate crimes,^37^ and the introduction of antitransgender legislation restricting access to health care and reversing protections prohibiting discrimination in health care based on gender identity. These examples highlight how sociocultural and political contexts may shape experiences of IBB among youth. Efforts to address IBB-associated health outcomes among Black transgender individuals must consider the multiple layers of oppression due to racism and transphobia. Furthermore, as data reflect the experiences of youth of race-based bullying in schools, prevention efforts should empower young people to engage in prosocial, antiracist behaviors with their peers, and schools should incorporate healing-centered practices that recognize racial trauma.

### Limitations

Our findings must be interpreted within the context of several study limitations. Data collection took place in urban neighborhoods in a single city; thus, findings may not generalize to adolescents in other geographic contexts. Missing data due to both survey opt-out and/or school absence and nonresponse to IBB questions could have biased findings. While youth have firsthand knowledge of IBB that may be unknown to others, the stigma of bullying may make youth reluctant to disclose being their experiences with IBB or IBB perpetration. Both forms of IBB were examined with single yes/no items for each stigmatized identity. More detailed assessment of these experiences, including of the frequency, severity, and perpetrator of IBB, is needed. Small subsample sizes required us to collapse across categories (eg, other collapsed a number of racial/ethnic identities) and precluded us from examining specific combinations of IBB. Future examinations incorporating larger samples are needed for a more nuanced intersectional examination of experiences of IBB and IBB perpetration. Finally, because of this study’s cross-sectional design, we are unable to draw directional conclusions from these data. While IBB itself may not directly lead to violence exposure or negative health or mental health, youth with exposure to IBB may be more likely to experience these outcomes because of structures of racism, homophobia, and other forms of identity-based discrimination.

## Conclusions

Despite a widespread increase in research on IBB, particularly race-based discrimination, studies that attend to multiple aspects of one’s social identity remain limited. This study found nuanced association patterns across types of IBB that factor into a number of health outcomes. Results encourage development of prevention programs that address bullying based on multiple marginalized identities. Policies and practices that address systemic racism and are attuned to the lived experiences of minority youth are imperative to support marginalized youth in clinical, school, and community-based settings.

## Notes

Supplement. **eTable.** Patterns of Experiences of IBB and IBB Perpetration Endorsed by Participants
