# Supplementary material for: Exploration of Experiences and Perpetration of Identity-Based Bullying Among Adolescents by Race/Ethnicity and Other Marginalized Identities
Source: JAMA Netw Open. 2021 Jul 23;4(7):e2116364. doi: 10.1001/jamanetworkopen.2021.16364 (PMC8303093; doi:10.1001/jamanetworkopen.2021.16364)
Supplement: Supplement. — eTable. Patterns of Experiences of IBB and IBB Perpetration Endorsed by Participants [file jamanetwopen-e2116364-s001.pdf]

Supplemental Online Content

Galán CA, Stokes LR, Szoko N, Abebe KZ, Culyba AJ. Exploration of experiences and perpetration of identity-based bullying among adolescents by race/ethnicity and other marginalized identities. *JAMA Netw Open.* 2021;4(7):e2116364. doi:10.1001/jamanetworkopen.2021.16364

eTable. Patterns of Experiences of IBB and IBB Perpetration Endorsed by Participants

This supplemental material has been provided by the authors to give readers additional information about their work.

© 2021 Galán CA et al. *JAMA Network Open.*

eTable. Patterns of Experiences of IBB and IBB Perpetration Endorsed by Participants

Race, ethnicity, or national origin	Religion	Sexual orientation	Gender	Physical or mental disability	Immigration Status	Other Reason	Number reporting this combination of IBB victimization[a]	Number reporting this combination of IBB perpetration[a]
							2434	2631
						■	616	445
■							269	169
	■						134	109
		■					103	47
			■				82	63
				■			37	47
					■		36	29
		■				■	27	0
■						■	24	0
				■		■	15	0
■					■		15	0
		■	■				12	0
		■	■			■	9	0
	■					■	9	0
■		■					8	0
		■		■			7	0
			■			■	7	0
		■		■		■	6	0
■			■				6	0
■	■						6	0
■	■				■		5	0
■	■	■	■	■	■	■	0	9

[a] Patterns endorsed by <5 participants are not included in the table.

© 2021 Galán CA et al. *JAMA Network Open.*
